# Exploring tailored virtual emotion regulation approaches for individuals with emotional eating

**DOI:** 10.1186/s40337-023-00856-2

**Published:** 2023-08-12

**Authors:** Aranka Dol, Lisette van Gemert-Pijnen, Lysanne M. Schwartz, Hugo Velthuijsen, Christina Bode

**Affiliations:** 1 Department of Psychology, Health and Technology, University of Twente, De Zul 10, 7522 NJ Enschede, The Netherlands; 2 Research Group New Business & ICT, Hanze University of Applied Sciences, Groningen, The Netherlands

**Keywords:** Emotional eating, Emotion regulation, Personalized coaching, Body scan, Positive reframing

## Abstract

**Background:**

Emotional eating is a complex problem fostering obesity and resulting from maladaptive emotion regulation. Traditional behavioural weight loss interventions have shown insignificant effect. They can be improved by targeting the specific needs of individuals with emotional eating.

**Objective:**

The current study explored a tailored online approach with the aim to positively influence affect (positive and negative) and emotion regulation by applying one of three exercises: body scan, opposite action, and positive reappraisal.

**Design:**

An embedded mixed-method design (questionnaire data (t0, t1, t2) and perceived usefulness of exercises in t2) was used to evaluate the effects of a two-week online quasi-experimental pilot study.

**Subjects/setting:**

In total, 80 participants with self-reported emotional eating difficulties (DEBQ-E; M_*emo*_ = 3.48, SD = .64, range 1.62–4.92) finished baseline measurements; 15 completed the intervention. The study sample was predominantly female (95%), from 18 till 66 (M_*age*_ = 38,0 ± SD = 14.25).

**Results:**

Participants reported that the exercises helped them to pay attention to their physical sensations, and to see positive aspects in negative matters. The exercises were considered difficult by the participants, with too little explanation, and dull, due to minor variation. The observed changes revealed small, and moreover, not significant improvements of the three exercises on positive and negative affect and overall emotion dysregulation. Although the quantitative results did not reach significance, the qualitative data highlighted which aspects of the tailored exercises may have contributed to mood and emotion regulation outcomes. A notable observation in the present study is the substantial dropout rate, with the number of participants decreasing from 80 at baseline (T0) to 15 at the post-intervention stage (T2).

**Conclusions:**

Future studies should identify tailored online exercises in emotion regulation skills in more detail and explore the contexts in which they are most effective in a personalized virtual coach virtual coach to be developed for individuals with emotional eating. Given the high dropout rate, more emphasis should be given to a proper presentation of the exercises, as well as more explanation of their usefulness and how to perform them.

## Abstract (plain language summary)

Emotional eating is eating in response to negative emotions, and is problematic because it may lead to overweight, depression, and low self-image. People with emotional eating behaviour have difficulty regulating emotions and need mental healthcare but may feel too ashamed to seek help. Moreover, healthcare is not always available at the time of need. Our goal in this research project is to develop a virtual coach application that is available 24/7. This study examined what users of such an application would think of a series of online exercises that can help you recognize and take control of your own negative emotions. We wanted participants to tell us what they thought of the exercises. We also measured whether performing the exercises had an effect on how a person felt (well-being) and whether their skills in handling their own emotions improved.The outcome was that participants found the exercises insightful, but that completion was dull. It also revealed that the measured effects were small.

## Introduction

In 2019, half the Dutch population (50.1%) of 18 years and older were overweight (BMI > 25) and 14.7% were obese (BMI > 30) [1]. Early research by Ganley [2] states that overweight and obese individuals (60% or more) struggle with emotional eating behaviour; the tendency to overeat in response to negative emotions, such as anxiety or irritability [3]. Emotional eating can occur regardless of satiation or hunger sensations and thereby can increase an individual’s caloric intake and foster obesity [4]. In turn, obesity increases the risk for other chronic conditions such as arthritis, cancer, diabetes, heart disease, high cholesterol, and hypertension [5]. Therefore, there seems to be a need for interventions effectively targeting emotional eating.

### Emotional eating behaviour

For individuals with emotional eating behaviour, eating and giving in to binge eating and or overeating, is a natural coping mechanism to deal with negative emotions [6, 7]. Individuals with emotional eating suffer from emotion dysregulation [8–11] limited awareness of their body’s internal signals, resulting in a diminished understanding of bodily sensations [12] and an elevated level of alexithymia, which refers to the difficulty in recognizing and describing one’s own emotions [13, 14].

### Traditional weight management

Until now, traditional behavioural weight loss interventions have demonstrated only little efficacy in reducing emotional eating [15]. This is likely because these interventions hardly give attention to the specific needs of individuals with emotional eating [16–18] such as using food for emotion regulation [15]. Emotion regulation is broadly defined as the extrinsic and intrinsic processes responsible for monitoring, evaluating, and modifying emotional reactions, especially their intensity and duration, to accomplish a personal goal [19]. When exploring the concept of emotion regulation, in the context of emotional eating, the act of overeating is commonly assumed to be a maladaptive emotion regulation strategy in itself (e.g., people overeat to regulate negative affect) [8, 9, 11].

However, findings propose that it is not the experience of negative affect, but rather a maladaptive regulation of the negative affect that is responsible for the overeating [20]. Accordingly, a longitudinal study among adolescents [20] found that a lack of emotion regulation abilities was associated with more emotional eating and subsequently could predict obesity [13, 21, 22]. Thus, it seems that it could be beneficial to assess the emotion regulation ability of participants to improve the tailoring of future online interventions for individuals with emotional eating behaviour.

Regrettably, the current provision of care falls short in adequately addressing the needs of individuals who engage in emotional eating. Individuals with emotional eating often feel a perceived disconnect from healthcare services. They experience feelings of shame regarding their eating issues [23] and will not readily seek care [23–26]. They tend to keep their problems related to emotional eating to themselves. They do not share with their GP (General Practitioner) that they have a problem with eating behaviour [54], thus, a considerable proportion of individuals with emotional eating remain invisible to the outside world [27–29]. If they do reach out to a GP for assistance, they are unlikely to directly address their eating problem, but focus on the secondary effects of obesity, such as diabetes or joint problems [30]. Consequently, there is minimal likelihood that the eating problems will be acknowledged or accurately diagnosed [27, 31].

### Virtual coaching

In an attempt to spark this advancement in the treatment of individuals with emotional eating, a few recent studies have looked at the effect of a virtual coach, which tailors exercises to specific needs of individuals with emotional eating [15, 30, 32–34]. For example, Dol et al. [35] aimed to develop a personalized coach, which tailors exercises to specific needs of individuals with emotional eating. In the study of Dol et al. [35], they identified three needs of individuals with emotional eating in the context of experiencing food cravings, (1) a need for insight in how to recognize and differentiate bodily signals associated with either emotions or food cravings, (2) a need for action plans that inspire alternative behaviour, when self-control difficulties are experienced during food cravings, and (3) a need for emotion regulation strategies that help to effectively regulate emotions like agitation, stress and anger.

With the desire to meet the aforementioned needs and to provide guidance with deployment of a personalized virtual coach, a measurement tool was selected. The most used instrument to measure difficulties in emotion regulation (i.e., emotion dysregulation) is the Difficulties in Emotion Regulation Scale [36]. The original DERS consists of 36 items distributed across six subscales measuring: (1) difficulties with accepting negative emotions or responding negatively to them (nonacceptance subscale), (2) difficulties with concentrating on and achieving a task when experiencing negative emotions (goals subscale), (3) difficulties with understanding and knowing the specific emotion one is experiencing (clarity subscale), (4) difficulties with being aware of or attending to one’s own emotional responses (awareness subscale), (5) difficulties with controlling impulsive behaviour when experiencing negative emotions (impulse subscale), and (6) an individual’s belief that there are limited options to effectively regulate one’s emotions once upset (strategies subscale). When placing the three needs of individuals with emotional eating in the context of the DERS subscales, the needs seem to relate best to three of the six subscales (Table 1).

**Table 1:** Needs of individuals with emotional eating related to DERS-SF* subscales

The need for…	Difficulty with…	DERS* subscale	Tailored exercise
Insight in bodily sensations	Being aware of or paying attention to emotional responses	*Awareness*	Body scan
Action plans that inspire alternative behaviour when self-control difficulties are experienced	Inhibiting impulsive behavioural responses	*Impulse*	Opposite action
Emotion regulation strategies to effectively regulate emotions, such as agitation, stress & anger	Effectively regulating one’s emotions once upset	*Strategies*	Positive reframing

*Difficulties in Emotion Regulation Scale [37]

Firstly, the need for insight in bodily sensations to be able to recognize sensations associated with either emotions or food cravings [35] seems to reflect difficulties with being aware of or paying attention to emotional responses. Subsequently, it was expected that this need relates to items of the awareness subscale which measures awareness of one’s own emotions.

Secondly, the need for action plans that inspire alternative behaviour when self-control difficulties are experienced seems to reflect difficulties with inhibiting impulsive behavioural responses. Therefore, it was expected that this need relates to items of the impulse subscale.

Finally, the need for emotion regulation strategies to effectively regulate negative emotions (e.g., agitation, stress, and anger) relates to difficulties with effectively regulating one’s emotions once upset. It was expected that this need relates to items of the strategies subscale. Thus, as three of the six subscales of the DERS (i.e., awareness, impulse, and strategies) seem to reflect the three identified needs of individuals with emotional eating, it is worthwhile to explore if the DERS can be used as an instrument to screen individuals with emotional eating in the context of a virtual coach application.

To find out whether the needs of individuals with emotional eating as described above could be addressed with the deployment of the personalized virtual coach, three exercises were selected that could have an effect on both positive and negative affect and emotion regulation (last column of Table 1).

#### Body scan

Firstly, to tailor to the first need (i.e., the need for insight in bodily sensations), an observational exercise described in the study protocol of Brevers et al. [37] can be used. The observational exercise is aimed at promoting intuitive eating (i.e., attuned, or mindful eating) in obese patients, by encouraging them to become aware of hunger and inner body sensations that might emerge from their emotions [38]. Participants are asked to indicate (before eating): (1) the intensity of hunger (on a 10-point scale), (2) activated or deactivated bodily regions (13 regions in total), and (3) the most relevant affect state.

Particularly step two, could be of interest in the current study as it is focused on being aware of sensations in different regions of the body. As the current study focused initially on exploring the effect of the tailored exercises regardless of the context (e.g., food cravings or eating), the first step of the observational exercise was excluded. Furthermore, according to the previously described Levels of Emotional Awareness Theory, individuals who refer to bodily sensations when expressing their emotions (level 1) have difficulties with explicitly stating the emotion that they are feeling (level 3). This difficulty was kept in mind when step three was added to the observational exercise for the current study.

#### Opposite action

Secondly, to tailor to the second need of individuals with emotional eating (i.e., the need for action plans that inspire alternative behaviour, when self-control difficulties are experienced), a skills exercise from the dialectical behavioural therapy (DBT) called “Opposite Action” (OA) can be used. This exercise is focused on helping individuals with identifying action urges that are associated with their emotions (e.g., anxiety motivates people to avoid the situation) and subsequently invites them to act the opposite (e.g., approach the situation) [38]. Findings of Ben-Porath et al. [38] suggested that repeated practice of the OA skill yields improvements regarding impulsivity. A pilot study of Rizvi et al. [39] showed related results. Over the course of a two-week trial among individuals with borderline personality disorder and substance use disorder, repeated practice with an OA exercise resulted in a significant decrease in both emotional intensity and urges to use drugs after each session. During each session participants were asked to indicate: (1) the emotion that they were experiencing, (2) the action urge, and (3) an opposite action from a list of emotion-specific options. In the current study the use of an opposite action exercise similar to the exercise developed by Rizvi et al. [39] was proposed.

#### Positive reframing

Lastly, to tailor to the third need of individuals with emotional eating (i.e., the need for emotion regulation strategies to effectively regulate emotions such as agitation, stress, & anger), a cognitive reappraisal exercise can be used [40]. Cognitive reappraisal can be defined as reinterpreting the meaning of emotional stimuli and with that changing the emotional response [41]. Findings in both laboratory and naturalistic settings show that cognitive reappraisal yields improvements in self-reported emotional states, such as anger [42], disgust [43], anxiety [44, 45], and perceived stress [46]. When comparing three different cognitive reappraisal techniques (a. positive reframing, b. self-distancing, and c. temporal distancing) regarding their effect on well-being, all three techniques yielded similar effects [41]. Where the positive reframing technique required individuals to find the positive aspects in the negative or stressful event, the self-distancing technique required individuals to visualize themselves in the negative or stressful situation from a third-person perspective and the temporal distancing technique required individuals to look back at the negative or stressful event one, five and ten years from now. The latter two techniques (i.e., self-distancing and temporal distancing) can be seen as more difficult as they require a greater perspective shift than the positive reframing technique [41]. Thus, in the current study the use of the positive reframing exercise was proposed.

The current study explored the experiences of individuals with emotional eating about the used online tailored exercises to gain a better understanding of how the exercises may have produced the effects found from the client/user perspective.

Furthermore, the effects of the three online exercises tailored to the three specific difficulties in emotion regulation, on affect, and on overall emotion dysregulation were explored. The study hypothesized that each exercise would decrease specific difficulties in emotion regulation, due to the tailoring, and with that would increase positive affect, decrease negative affect, and decrease overall emotion dysregulation. The study also hypothesized that based on three varying emotion regulation difficulties measured by DERS, three distinct groups of individuals with emotional eating could be distinguished. Thus, to gain more knowledge on how to meet the needs of the emotional eater with the use of a personalized virtual coach, we formulated the following research questions.

**Main research question:**

- To what extent and in which way do experiences of individuals with emotional eating about three online tailored exercises, contribute to a better understanding of the hypothesized changes in affect and emotion dysregulation?
- Research question 1: How do participants perceive the usefulness and feasibility of the three tailored exercises?
- Research question 2: what kind of suggestions for improvement do participants have experiencing the three tailored exercises?
- Research question 3: What is the effect of three online tailored exercises on positive affect, negative affect, and overall emotion dysregulation?

## Methods

### Design

This pilot study used an embedded mixed-method design [47], to evaluate the effects of a two-week online quasi-experiment with three groups. Qualitative data was collected to gain a better understanding of the measured effects of the exercises. Simultaneously, quantitative data was collected to explore the effects of three tailored exercises on affect (negative and positive) and emotion dysregulation.

#### Participants

Using convenience sampling, 80 participants with self-proclaimed emotional eating difficulties have been recruited. The questionnaire on eating behaviour DEBQ-E [48] was filled in at T0 (pre-intervention; *M*_*emo*_ = 3.48, SD = 0.64, range 1.62—4.92). Measuring with the DEBQ-E allowed us to properly check whether we reached the target population. Participants were recruited through sending out an invitation: (1) to clients of two dietitian practices with a treatment program for people with overweight or obesity (Centrum Leefstijl en Zorg, CleZ, and DieetZorg), (2) to clients of an outpatient clinic, dietetics department (Nij Smellinghe Hospital); (3) to participants from the study of Dol et al. [35], who indicated to be willing to participate in follow-up research; (4) through an online newsletter from the Dutch Patient Association for Eating Disorders (WEET), and (5) on various social media outlets (i.e., a private Facebook group “Emotie eten”; an online forum for people with eating difficulties “Proud2Bme”; story mentioning’s on the Instagram Pages of six Dutch eating coaches). The following inclusion criteria were used: (1) age of 18 years or older; (2) experience with emotional eating difficulties (DEBQ-E); (3) sufficient knowledge of the Dutch language. The participants were not paid or otherwise rewarded.

### Materials

#### Emotional eating behaviour

Emotional eating behaviour was assessed using the Emotional Eating scale of the Dutch Eating Behaviour Questionnaire (DEBQ-E) [48]. The scale contains 13 items about emotional eating with four items about dealing with eating in response to diffuse emotion and nine items about dealing with eating in response to clearly labeled emotions. Each item was rated on a 5-point Likert scale ranging from 1 “never” to 5 “very often”. Scores on the DEBQ-E were comprised by dividing the sum of the items scored by the total number of items [16]. The emotional eating scale showed good internal consistency in both the original validation (α = 0.94) and in the current study (α = 0.86).

#### Positive and negative affect

Positive and negative affect were measured using the International Positive and Negative Affect Schedule Short Form (I-PANAS-SF) [49]. The period over which the participants had to give their self-assessment was "the past week".

It is a 10-item questionnaire that consists of five positive and five negative emotions. Each emotion was rated on a five-point Likert scale ranging from 1 “very slightly” to 5 “very much”. The positive emotions are alert, inspired, determined, attentive, and active. The negative emotions are upset, hostile, ashamed, nervous, and afraid. The Dutch translations of these emotions were derived from a Dutch version [50] of the original 20-item PANAS [51]. Previous research showed that the I-PANAS-SF had acceptable psychometric properties [49]. Similarly, the Dutch version of the PANAS showed sufficient validity and reliability [50]. In the current study, the internal consistency of the positive affect scale was α = 0.77 at T0 (n = 80), α = 0.68 at T1 (n = 26), and α = 0.62 at T2 (n = 15). For the negative affect scale, the internal consistency was α = 0.63 at T0 (n = 80), α = 0.69 at T1 (n = 26), and α = 0.82 at T2 (n = 15).

#### Emotion regulation difficulties

Emotion regulation difficulties were measured using the Difficulties in Emotion Regulation Scale Short Form (DERS-SF) [51]. This scale contains 18 items and measures emotion regulation difficulties (i.e., emotion dysregulation) across six dimensions: non-acceptance, goals, clarity, awareness, impulse, and strategies. Items were rated on a five-point Likert scale ranging from 1 “almost never” to 5 “almost always”. The DERS-SF showed excellent psychometric properties in its original validation. Cronbach’s alpha coefficients for all six DERS-SF subscales ranged from 0.79 to 0.91 [52]. In the current study, the internal consistency of the three subscales used for screening were: α = 0.78 at T0 (n = 80), α = 0.79 at T1 (n = 26) and α = 0.80 at T2 (n = 15) for the awareness subscale; α = 0.83 at T0 (n = 80), α = 0.49 at T1 (n = 26) and α = 0.89 at T2 (n = 15) for the impulse subscale, and; α = 0.71 at T0 (n = 80), α = 0.74 at T1 (n = 26) and α = 0.75 at T2 (n = 15) for the strategies subscale.

#### Qualitative data

At post-intervention (T2) the participants were asked five open-ended questions about their experiences with the exercise performed.

*“Which aspects of the exercises did you find useful?”, “Which aspects of the exercise did you not find useful?”, “Which (new) insights or skills did you acquire by practicing with the exercise?”, “How would you adapt the exercise so it would be able to help you better?”* and* “Is there anything else you would like to share with us?”.*

### Procedure

#### Ethics

This study was approved by the BMS Ethics Committee of the University of Twente (file number: 200091).

#### Study procedure

An overview of the study procedure is presented in Table 2. Before participation, participants were informed about the research aim and asked if they wanted to take part in the current study. Participants were enrolled on a voluntary basis and after reading the information sheet and consenting to participate. Then, participants filled out demographic questions, the DEBQ-E, the I-PANAS-SF and the DERS-SF. Based on their scores on the awareness, impulse and strategies subscale of the DERS-SF, participants were assigned to one of the three tailored exercises, as described in paragraph *Tailored exercises* on the next page.

**Table 2:** Study procedure

	Study period			
	Inclusion	Screening	Post-allocation	
*Timepoint*		T0	T1 (1wk)	T2 (2wk)
Enrolment				
Study invitation to participant	×			
Online informed consent	×			
Check inclusion criteria		×		
Assessments				
Demographic questions^a^		×		
BMI, kg/m^2^		×		
Questionnaires				
* DEBQ-E*		×		
* I-PANAS-SF*		×	×	×
* DERS-SF*		×	×	×
Open questions				×
Allocation^b^		×		
Tailored exercises				
Body scan (group A)^c^	**→**			
Opposite action (group B)^d^	**→**			
Positive reframing (group C)^e^	**→**			

BMI, body mass index; DEBQ-E, Dutch Eating Behaviour Questionnaire; I-PANAS-SF, International Positive and Negative Affect Schedule Short Form; DERS-SF, Difficulties in Emotion Regulation Scale Short Form

^a^Questions about gender, marital status, living situation, highest level of education, employment status, age, length (in m), weight (in kg) and number of consultations with dietitians

^b^Based on the DERS-SF awareness, impulse, and strategies subscale scores

^c^Assigned if awareness subscale score ≥ 9

^d^Assigned if awareness subscale score > 9 AND impulse subscale score ≥ 9

^e^Assigned if awareness AND impulse subscale score > 9 AND strategies subscale score ≥ 9, OR if awareness, impulse, AND strategies subscale scores > 9

#### Assignment of participants

Participants in the current study were assigned to one of three groups based on their scores on the three DERS-SF subscales (i.e., awareness, impulse, and strategies) at baseline (T0). The group receiving the body scan exercise was called group A. The group that received the opposite action exercise was called group B and the group that received the positive reframing exercise was called group C.

##### Group A—body scan

Participants who scored high on the awareness subscale (score ≥ 9; that is, the respondent scored 3 or higher on all 3 items of the subscale), having difficulties with being aware of or paying attention to emotional responses, were assigned to a group with participants that received the body scan exercise.

##### Group B—opposite action

Participants who scored low on the awareness subscale (score > 9) AND high on the impulse subscale (score ≥ 9) were assigned to a group with participants that received the opposite action exercise.

##### Group C—positive reframing

Lastly, participants who scored low on both the awareness AND impulse subscales (scores > 9) AND high on the strategies subscale (score ≥ 9) OR scored low on all subscales (all scores > 9) were assigned to a group with participants that received the positive reframing exercise.

#### Course of the study

Participants were asked to practice (± 15 min) every other day with the assigned exercise for two weeks. The duration of the current study protocol is in line with the typical duration of daily diary studies; one to four weeks [53]. Participants were provided access to the exercise within the online survey environment through email on alternate days. The exercises were accompanied by instructions.

In addition to baseline measurements (T0) of the I-PANAS-SF and DERS-SF, measurements were conducted after one week at mid-intervention (T1), and after two weeks at post-intervention (T2). The post-intervention questionnaire also included five open questions about their experience with the assigned exercise *(Methods* > *Materials* > *Qualitative data*).

### Tailored exercises

Matching the DERS sub-scales *awareness*, *impulse*, and *strategies*, three different exercises were selected: body scan, opposite action, and positive reframing. They were presented to the individual participants via Qualtrics, an online survey tool [54]. Participants conducted one of three exercise which was tailored to their emotion regulation difficulty. Written instructions were attached to the exercises (Appendix 1, 2, and 3).

#### Body scan

The *body scan exercise* was aimed at encouraging individuals with emotional eating to become aware of and pay attention to inner body sensations that may have emerged from their emotions. The body scan exercise was derived and adapted from an observational exercise described in the study protocol of Brevers [37] and required participants to indicate per bodily region (13 in total) whether it felt pleasant (including neutral) or unpleasant based on a topographical self-report method [55]. Subsequently, participants were challenged to think about what could be causing the pleasant/unpleasant sensations in their body, with answer options ranging from: (1) bodily/physiological processes (e.g., hunger, thirst, muscle soreness, disease symptoms), (2) an emotion, or (3) other. A text entry box was provided for each answer option to offer participants different answering options. These answers were not stored.

#### Opposite action

The *opposite action exercise* is aimed at helping individuals to identify action urges associated with their emotions (e.g., anxiety motivates people to avoid the situation) and inviting them to act the opposite (e.g., approach the situation) [33]. The opposite action exercise was derived and adapted from the studies of Rizvi et al. [39], and Ben-Porath et al. [38]. During each session participants were asked to describe or identify: (1) a situation on which they wanted to reflect, (2) their behaviour in that situation, (3) an emotion that could have motivated their behaviour, and (4) an opposite action that could have helped to regulate the emotion. Lastly, participants were encouraged to apply the opposite action in practice and observe changes in their emotional experience. The opposite action exercise was accompanied by a list of emotions, emotion-specific action urges and opposite actions drafted using the DBT Skills Training Manual (Emotion Regulation Handout 11) [56] and the Emotions Motivate Actions Information Handout of Psychology Tools [57]. The latter was also used to design a short introductory exercise (in which the participant could "drag" behavioural description to the appropriate emotion), which participants could do to practice with linking action urges to specific emotions before starting with the exercise.

#### Positive reframing

The *positive reframing exercise* was aimed at encouraging individuals with emotional eating to find positive aspects in negative or stressful events as a strategy to regulate emotions such as anger and stress. The positive reframing exercise was derived and adapted from the study of Ranney et al. [41] and required participants to: (1) describe an unpleasant event on which they wanted to reflect, (2) describe their thoughts and feelings evoked by the unpleasant event, (3) Lastly, participants were challenged to think of other possible explanation(s) for what happened, find positive aspects in the unpleasant event, find things they could learn from the unpleasant event, and/or describe how this event might be helpful for them.

### Data analysis

To explore the experiences of individuals with emotional eating about the online tailored exercises, the written answers to the five open questions were collected from an SPSS (version 25) file and imported into a Microsoft Office Word document, sorted per intervention group. Thematic coding [58] was used to facilitate the analysis of the answers to the open question. All meaningful fragments, consisting of either a sentence, a part of a sentence or a combination of sentences, were coded by LS into overarching themes by analyzing the underlying meaning of each fragment. An iterative process facilitated the adjustment and fine-tuning of the final fifteen coding schemes. The final coding schemes were peer-reviewed by AD. For all coding schemes, an acceptable inter-rater reliability (LS and AD; range 0.75 to 1.00) was reached after the first round of coding. The qualitative data about the experiences of individuals with emotional eating with the online tailored exercises was used to help interpret the found effects.

To explore the effect of the three tailored exercises on positive affect (PA) and negative affect (NA), measured with the I-PANAS-SF, and on emotion dysregulation, measured with the DERS-SF, summed PA and NA scores, and DERS-SF total and subscales scores at baseline (T0), mid-intervention (T1), and post-intervention (T2) were compared using a paired t-test. In case of small intervention groups and not normally distributed data, a Wilcoxon signed-rank test was used instead of a paired t-test.

## Results

### Participants’ baseline characteristics

A total of 80 participants were enrolled in the study and completed the questionnaires at baseline. Of the 80 participants, 41 received the body scan exercise (group A), five received the opposite action exercise (group B) and 34 participants the positive reframing exercise (group C), according to the study procedure. Participants’ baseline characteristics are provided in Table 3. The table presents the means and the standard deviations of the variables. The mean score on DEBQ-E is highly similar to those of the norm-group of obese women with ages between 21 and 40 years [59].

**Table 3:** Participants’ baseline characteristics

Characteristic	T0 (*n* = 80)	T1 (*n* = 33)	T2 (*n* = 15)
Age, years, mean ± SD (range)	38 ± 14.3 (18–66)		
Female sex, n (%)	76 (95.0)		
BMI, kg/m^2^, n (%)			
> 25	24 (30.0)		
25–30	17 (21.3)		
> 30	39 (48.8)		
DEBQ-E, mean ± SD (range)	3.48 ± 0.64 (1.62–4.92)		
No consultation with dietitian, n (%)*	35 (43.8)		
Received body scan exercise, n (%)	41 (51.3)	22	8
Received opposite action exercise, n (%)	5 (6.3)	3	2
Received positive reframing exercise, n (%)	34 (42.5)	22	5

SD, standard deviation; BMI, body mass index; DEBQ-E, Dutch Eating behaviour Questionnaire-Emotional.

*We asked participants whether they ever consulted a dietitian

At mid-intervention, 47 of the 80 participants had dropped out. In total, 15 of the 80 participants (19.0%) completed post-intervention measurements, eight participants from group A, two participants from group B and five participants from group C. One-way ANOVA calculations showed no significant differences between the completers and the dropout groups on any socio-demographic characteristics and questionnaires at baseline.

This paragraph summarizes the found effects of the exercises on affect and emotion dysregulation. As expected with the tailored assignment based on the first measurement, ANOVA calculations showed significant differences at baseline on all emotion related variables (Table 4). However, Tukey post hoc calculations reveal that only two of three intervention groups significantly differed from each other on all emotion related variables, namely group A and C. Participants from group C experienced significantly more positive affect (*p* = 0.002) and significantly less negative affect (*p* = 0.048), emotional eating (*p* = 0.009) and emotion dysregulation (*p* > 0.005), compared to participants from group A. Contrastingly, participants from group B did not significantly differ from group A on any of the emotion related baseline variables. As the assignment of tailored exercises was based on emotion dysregulation scores at baseline, this may explain the small number of participants in group B (n = 5), compared to group A (n = 41) and C (n = 34).

**Table 4:** Baseline differences between intervention groups

Calculations/Variables	Emotional eating	Emotion dysregulation	PA	NA
	*M/SD*	*M/SD*	*M/SD*	*M/SD*
ANOVA				
Group A (SD) (n = 41)	47.49 (7.87)	56.07 (10.44)	14.59 (3.26)	13.83 (2.64)
Group B (SD) (n = 5)	49.80 (5.97)	53.00 (8.22)	17.40 (2.88)	15.20 (1.79)
Group C (SD) (n = 34)	41.88 (8.21)	39.76 (8.25)	17.12 (3.00)	12.24 (3.19)
F-value	5.516	28.16	6.745	4.150
* p*-value	0.006*	> 0.005*	0.002*	0.019*
Post hoc				
Group A-C (SE), *p*	5.61 (1.84), 0.009*	16.31 (2.19), > 0.005*	− 2.53 (0.73), 0.002*	1.59 (0.66), 0.048*
Group B-C (SE), *p*	7.92 (3.80), 0.100	13.24 (4.53), 0.012*	0.28 (1.50), 0.981	2.96 (1.37), 0.084
Group A-B (SE), *p*	− 2.31 (3.76), 0.812	3.07 (4.48), 0.772	− 2.81 (1.48), 0.146	− 1.37 (1.35), 0.571

PA, positive affect; NA, negative affect; M, mean; SD, standard deviation; SE, standard error

*significant at a 0.05 significance level

### Research question 1: How do participants perceive the usefulness and feasibility of the three tailored exercises?

This paragraph provides a summary of the participants’ positive and negative experiences, as well as their suggestions for improving the presentation and instructions for performing the exercises. In line with the Research Questions, we will discuss the qualitative results first. Given our aim to develop a virtual coach, we consider participants’ experiences and opinions to be crucial in shaping our approach.

#### Body scan

Six of the eight remaining participants in group A who practiced with the body scan exercise thought that the exercise could help them pay attention to their physical sensations (*“Thinking about what you feel and where, instead of just going on.”* (BS5^1^). They found it particularly helpful that the exercise asked them to determine if a sensation was either a physical or emotional response. One participant found the provided list of emotions helpful.

Three participants mentioned that with the exercise they were able to take a step back and incorporate relaxation into their day *“Take a break or relax sooner.”* (BS3). Furthermore, five mentioned that they learned to observe their physical sensations (*“That you feel things sometimes if you pay attention to it.”* (BS5)), or their emotions. Other participants got insights into what might cause their physical discomfort. The answers of two participants were related to poor self-care (*“[Taking a step back] this is something that I regularly fail to do because of workload or busyness in the family. My chronic back pain and muscle complaints are probably related to this.”* (BS4)). One answer was related to the impact of negative emotions: *“That negative emotions also can lead to a lot of uncomfortable bodily sensations.”* (BS8).

However, five participants provided feedback about aspects of the body scan exercise they found less useful. One mentioned that they were unable to write down their physical sensations and identify its causes. Another participant mentioned that they had gained no new insights or skills from practicing with the body scan exercise. *“I have no idea. I have no clue how this exercise can help me anyway.”* (BS1). Another participant mentioned the need for more examples (*“It would be good to provide an example under “other.”* (BS8)). One participant shared: *“I thought it was pretty vague that I had to indicate whether I thought a body part felt good or not. My stomach never feels good, but what does it matter?”* (BS1). Lastly, one participant mentioned experiencing increased physical tension during the body scan exercise: *“It is always like this and the more I focus on my body the more it starts to feel tense.”* (BS1).

#### Opposite action exercise

In the group who practiced with the opposite action exercise (group B) the two remaining participants filled in the post-intervention questionnaires. One participant stated gaining insight about the interaction between behaviour and emotions: *“Become aware of your behaviour and which emotion it comes from and what you can do differently.”* (OAE1).

#### Positive reframing exercise

All five remaining participants in the group C reported that the exercise helped them to reflect either on what happened in specific situations or on their feelings (*“You will start thinking a little deeper, thinking more consciously about how you feel and why you feel that way.*” (PRE1)). Two participants emphasized reframing as a useful aspect of the exercise: *“Practicing with reframing forces you to see positive aspects in negative things. I noticed that this was a pleasant thing to do and think that I will apply this more often when I experience setbacks.”* (PRE2).

Three answers illustrated broadening of reflection skills: *“[I am] more aware of how I react in different situations.”* (PRE1), *“Paying attention to how you think and whether it can be different.”* (PRE5) and *“By sometimes waiting a bit more to hear what someone else has to say about the situation.”* (PRE3).

Lastly, two participants’ answers referred to gained insights on how to deal with negative events. One specifically mentioned the skill to recognize positive aspects in negative situations, where another mentioned the skill to reevaluate the situation by distancing oneself from the situation *“Take a step back from the situation for one moment […] and review it.”* (PRE4).

However, three participants felt that the recurrence of practice moments was too frequent: *“It is not every day that you encounter an unpleasant event, at one point I found it difficult to think of a situation.”* (PRE2). In line with this, one participant reported that the exercise was too extensive for some situations.

### Research question 2: what kind of suggestions for improvement do participants have experiencing the three tailored exercises?

#### Body scan

Two participants mentioned a general need for tips regarding emotion-regulation. One participant specifically mentioned the need for help with dealing with positive or negative feelings: *“[…] what helps with holding on to positive feelings or to release negative feelings in a healthy way.”* (BS3). Where one participant suggested adapting the schedule of the exercise to a set time point each day, another one suggested the opposite: *“I have done the exercise when I was resting, [or] had time for it. But in hindsight it would also have been useful to do this at any time in between, even at a hectic or emotional moment, to gain insight into how I physically react to my emotional state at that particular moment.”* (BS4).

The latter participant suggested collecting real time data at varying time points. Regarding the order of the exercise, one participant suggested to reverse the exercise by first identifying the emotional state and then naming the physical sensation that comes with it (*“So reverse the exercise, as it were.”* (BS4)). Three answers were related to supplementing existing parts of the body scan exercise, like adding more emotions to the list of emotions, providing examples under “other causes,” adding a picture of the lower back and shoulder blades. Other suggested adaptations were related to the schedule, order, and duration of the body scan exercise, and adding a guided body scan. One participant suggested to make sure that the exercise does not take too long, keeping it at 25–30 min maximum: *“That it [the body scan exercise] can take too long, which causes me to lose my attention and concentration.”* (BS6). The same participant emphasized the importance of human contact: *“It is also nice to just talk about the difficulty you experience. I do not have any family or friends to turn to. Mindfulness alone is not enough.”* (BS6).

#### Positive reframing

One participant experienced a lack of variation during the two-week training protocol (*“I would like to have a change in exercise, for example after the first week based on the results of the half-point evaluation, a different exercise.”* (PRE1)). Regarding the specific content of the exercise, another participant suggested giving examples that could help with answering the questions that prompt reflection.

One participant indicated that users with different levels of education should be able to participate in the exercise: *“I don’t know which target group [has] to be reached, but I do think that you should have at least an MBO-level (vocational education) if you want to be able to answer the questions”.* Lastly, one participant suggested supplementing instructions at the beginning of the exercise: *“Perhaps you should mention at the outset that you should take your time and do the test and the exercises in a relaxed manner.”* (PRE1).

### Research question 3: What is the effect of three online tailored exercises on positive affect, negative affect, and overall emotion dysregulation?

#### Effects of the tailored exercises on positive affect, negative affect, and emotion dysregulation

As positive affect scores were not distributed normally in both group A and C, as well as three of the DERS-SF subscale scores in group A (respectively Shapiro–Wilk test: T0_PA_ = 0.034, T0_A_ > 0.001, T2_A_ = 0.029, T0_I_ = 0.008, and T1_S_ = 0.033), and five of the DERS-SF subscales scores in group C (respectively Shapiro–Wilk test: T2_PA_ = 0.03, T0_A_ = 0.018, T0_C_ = 0.007, T1_C_ = 0.001, T0_I_ = 0.01, T1_I_ = 0.034, T2_I_ = 0.042, T0_N_ = 0.011, and T0_S_ = 0.017), the Wilcoxon signed-rank test was used for all calculations for group A and C (Table 5).

**Table 5:** Descriptive statistics of main outcome variables at baseline (T0) and two post-tests (T1 and T2) and results of Wilcoxon Signed Ranks Tests for 3 treatment groups

	T0			T1			T2			T0—T1		T0—T2	
	*N*	*M*	*SD*	*N*	*M*	*SD*	*N*	*M*	*SD*	*Z*	*p*	*Z*	*p*
Group A													
Positive affect	41	14.59	3.26	14	14.71	3.15	8	15.75	2.05	− 0.135	0.892	− 0.954	0.340
Negative affect	41	13.83	2.64	14	13.79	3.19	8	13.63	3.11	− 1.464	0.143	− 0.282	0.778
Awareness	41	10.73	1.67	14	10.43	2.50	8	10.75	2.31	− 1.813	0.070	− 1.633	0.102
Impulse	41	7.41	3.03	14	6.57	2.47	8	6.63	2.88	− 0.791	0.429	− 0.345	0.730
Strategies	41	8.41	2.41	14	8.43	2.14	8	8.13	2.75	− 0.810	0.418	− 0.690	0.490
DERS-SF total	41	26.56	4.92	14	25.43	4.50	8	25.50	5.01	− 1.699	0.089	− 0.986	0.324
Group B													
Positive affect	5	17.40	2.88	2	15.00	4.24	2	17.50	0.71	− 1.342	0.180	− 0.447	0.655
Negative affect	5	15.20	1.79	2	16.00	2.83	2	12.50	2.12	− 0.447	0.655	− 1.342	0.180
Awareness	5	5.40	0.55	2	6.00	0.00	2	5.00	0.00	− 1.414	0.157	0.000	1.000
Impulse	5	10.60	1.82	2	5.50	0.71	2	8.50	2.12	− 1.342	0.180	− 1.414	0.157
Strategies	5	9.60	3.29	2	6.50	3.54	2	11.00	2.83	0.000	1.000	− 1.000	0.317
DERS-SF total	5	25.60	5.32	2	18.00	4.24	2	24.50	4.95	− 1.000	0.317	− 1.000	0.317
Group C													
Positive affect	34	17.12	3.00	10	18.40	2.01	5	18.60	3.21	− 0.426	0.670	− 1.511	0.131
Negative affect	34	12.24	3.19	10	11.00	2.54	5	10.40	4.34	− 1.086	0.277	− 0.677	0.498
Awareness	34	5.88	1.53	10	5.50	1.72	5	5.80	1.79	− 0.408	0.683	− 1.342	0.180
Impulse	34	5.00	1.52	10	5.00	2.05	5	4.00	1.41	− 1.150	0.250	0.000	1.000
Strategies	34	5.91	1.88	10	6.20	2.44	5	5.20	1.10	− 0.499	0.618	− 0.816	0.414
DERS-SF total	34	16.79	3.91	10	16.70	4.69	5	15.00	3.00	− 0.818	0.413	0.000	1.000

M, mean; SD, standard deviation; Z, Z-score; DERS-SF, Difficulties in Emotion Regulation Scale Short Form

#### Positive affect, negative affect, emotion dysregulation

Firstly, in line with the study expectations, the results of a Wilcoxon signed rank test showed a small increase in positive affect scores for both group A (T0 = 14.59, T1 = 14.71, T2 = 15.75) and group C (T0 = 17.12, T1 = 18.40, T2 = 18.60) as well as a small decrease in negative affect scores for both group A (T0 = 13.83, T1 = 13.79, T2 = 13.63) and group C (T0 = 12.24, T1 = 11.00, T2 = 10.40). Also, emotion dysregulation scores, reflected by the DERS-SF total score, decreased over the two-week training protocol for group A (T0 = 56.07, T1 = 55.50, T2 = 54.63), as well as for group C after a minor increase at mid-intervention (T0 = 39.76, T1 = 40.40, T2 = 36.00). However, none of these results reached statistical significance.

#### Group A (body scan)

Furthermore, it was expected that participants in group A, who received the body scan exercise, would experience significantly less difficulties with being aware of or paying attention to emotional responses after the two-week training protocol, indicated by a significant decrease in awareness subscale scores. Although not significant, the results did reveal a marginally significant decrease in awareness subscale scores (*p* = 0.07) between baseline (T0 = 10.73) and mid-intervention (T1 = 10.43), which is in line with the expected direction in changes.

#### Group B (opposite action)

Because of the consistently small sample size in Group B during the entire two-week training protocol (T0 = 5, T1 = 2, T2 = 2), the quantitative results regarding the impact of the opposite action exercise on positive affect, negative affect, and emotion regulation difficulties are not further elaborated upon (Table 5).

#### Group C (positive reframing)

Lastly, it was expected that participants in group C, who received the positive reframing exercise, would experience significantly less difficulties with effectively regulating their emotions once upset, indicated by a significant decrease in strategies subscale scores. In contrast to the study expectations, the results showed no significant decrease in strategies subscale scores in group C throughout the two-week training protocol.

## Discussion

### Key findings

This study’s main aim was to explore a tailored online approach to influence affect (positive and negative) and emotion regulation by applying one of three exercises: body scan, opposite action, and positive reappraisal.

The results of this study demonstrate that online exercises to address emotion dysregulation are beneficial for hard-to-reach target groups. Participants expressed interest and willingness to participate in online treatment exercises. Online exercises might lower the threshold for individuals with emotional eating to seek help. Performing the exercises gave participants a sense of awareness about their own bodily sensations and negative emotions.

### User experiences participants

Considering the participants’ experiences about the usefulness and feasibility of the exercises the main finding was that they helped participants to pay attention to one’s own body. According to the participants it was a valuable experience to become aware of one’s physical sensations, and to examine what possibly caused them. Participants experienced it as insightful to take a brief time out, and to step away from the daily hassles, for a moment of rest. Performing the exercises pushed them to look for the positive aspects of an issue. It also helped them to reflect either on what happened in specific situations or on their feelings. From the given answers, the relevant reflection skills had been broadened.

The number of studies evaluating the effects of body scan is growing [60–63]. The literature indicates that the body scan helps with stress reduction. Performing exercises such as the body scan, enhances the awareness of emotional and cognitive events in the present moment. Individuals become capable of recognizing early signs of tension accumulation [64]. Individuals who experience elevated levels of arousal and stress are likely to encounter physiological symptoms, such as muscle tension. By attentively acknowledging these symptoms while maintaining a nonjudgmental mindset, it is possible to alleviate these physical reactions [65, 66].

Body scan is considered an accessible introductory exercise [65], but some participants experienced unpleasant physical sensations and were unable to interpret them. They were encouraged to notice and accept the sensations they experience openly and nonjudgmentally, but not everyone seemed capable of doing that. Participants did not know what to do with it. Due to the recognized limited awareness of their body’s internal signals in this group of individuals with emotional eating, leading to a reduced understanding of bodily sensations [12], special emphasis should be placed on teaching skills to enhance the recognition of these sensations.

Moreover, the mere act of being aware of physical sensations, such as tingling, numbness, itchiness, pulse, skin stretch, warmth, coolness, muscular stiffness, and vibration in the body, can elicit physical sensations. Tihanyi, Ferentzi and Köteles [67] describe this phenomenon as attention-related body sensations. In their research they explain that focused attention on the body can trigger (automatic) thoughts and judgments about the body or experienced sensations. These thoughts and judgments can activate negative emotions, such as shame or fear, which in turn can bring about changes in physiological sensations (e.g., muscle stiffness). This may explain why one of the participants from the current study experienced an increase in bodily tension while practicing with the body scan exercise. Other participants indicated a need for additional information about physical sensations and its potential causes (e.g., physiological, or psychological). Physiological changes, such as reduction in muscle tension, can be measured using sensor technology (muscle tension sensor) [68]. Disturbances in heart rate variability caused by stress can also be monitored by sensors in a wearable device, such as a watch [69]. The outcomes of those measurements can be fed back to the user via the virtual coach, who can pair their own experiences with the information generated by the virtual coach and thus deepen their insights.

The feedback regarding positive reframing was mostly positive, as it prompted participants to actively engage in thoughtful reflection on events and even identify positive elements within negative situations ("…forces you to see positive aspects in negative things"). On the other hand, the negative aspects of this exercise were primarily related to the repetitive nature of the task, with the absence of a specific trigger at times, which made it feel less necessary to perform the exercise.

Certain participants found the exercises to be monotonous, lacking variety in execution. They perceived them as too challenging and incomprehensible, with inadequate guidance on how to perform them. Some participants even considered the exercises to be pointless due to a lack of understanding of their purpose. Participants expressed that there was insufficient support to fully grasp the exercises, particularly finding the body scan exercise to be difficult.

Emotional eating behaviour is frequently accompanied by alexithymia, which refers to the difficulty in recognizing and describing one’s own emotions [13, 14, 70]. Individuals with underdeveloped skills in this area can benefit from psychoeducation to enhance their understanding of their own emotions [71–73].

The lack of clear instructions and limited online assistance demotivated users. Consequently, participants recommended the inclusion of examples and visual aids, such as pictures, to enhance comprehension and engagement.

The target group is fully capable of engaging in self-management exercises online, and, in addition, individuals with emotional eating often prefer this approach, partially due to feelings of shame [74–76].

Improved presentation and attractiveness of the exercises, along with providing sufficient and comprehensive instructions on their execution and potential experiences during the process, would have influenced their effectiveness on positive affect (PA), negative affect (NA), and emotion dysregulation.

#### Supplementing the body scan exercise

It may be the case that the body scan exercise is less appreciated since the current study did not consider the context of experiencing hunger or food cravings, in contrast to Dol et al. [35]. Participants should practice with the assigned emotion regulation exercises when not experiencing food cravings or hunger. However, it may be said that it can be difficult to recognize the difference between hunger and emotionally evoked bodily sensations. Murray and Vickers [77] describe various physical (e.g., empty/hollow feeling) and mental experiences (e.g., lack of concentration on task) of typical and extreme hunger alone. Hunger is controlled by gastrointestinal mechanisms. Hunger is the presence of stomach growls, stomach hunger pains, emptiness, focus on eating, and loss of energy [77], whereas emotionally evoked sensations are related to sudden cravings, physical or mental exhaustion, unaddressed stress, and the desire to relieve it. We believe that the existing body scan exercise gains added value when extended with knowledge about physical and emotional hunger.

### Effects of exercises

To improve the tailoring of future interventions for individuals with emotional eating, this study explored the effect of three online exercises, tailored to three specific emotion regulation difficulties, on affect (positive and negative) and overall emotion dysregulation within a sample of individuals with emotional eating. Based on their most prominent emotion regulation difficulty, participants received either the body scan, opposite action, or positive reframing exercise.

Knowledge and insights were gathered on the effects of the three tailored exercises on the DERS and PANAS scores, but the hypothesized effects were too small to be categorized as significant. The small effects were caused by the small number of participants in T1 and T2. There was also minimal allocation to Group B, so results could not be retrieved. High dropout rate may have caused the measured effects to be small.

It was expected that based on three varying emotion regulation difficulties, measured by DERS, three separate groups of individuals with emotional eating could be distinguished.

Analysis revealed that two groups of individuals with emotional eating (A. body scan and C. positive reframing) significantly differed on all emotion related variables at baseline. This finding emphasizes the importance and feasibility of developing tailored interventions for this group. However, considering participants’ feedback expressing their preference for different exercises, tailoring interventions may limit their access to a variety of options. To address this, it could be beneficial to offer a broader range of exercises that target a specific difficulty in emotion regulation.

A third group (B. opposite action), based on difficulties with inhibiting impulsive behavioural responses, could not be distinguished. Compared to the other groups, few people were found for this pattern (n = 5) and the pattern did not significantly differ from group A on any of the baseline variables and from group C only on one of four baseline variables.

Due to the particularly low sample size in group B throughout the two-week training protocol (${n}_{T0}$= 5, ${n}_{T1}$= 2, ${n}_{T2}$= 2), the current study was unable to describe scientifically relevant results for the opposite action exercises.

Subsequently, tailoring the future virtual coach based on this emotion regulation difficulty should be omitted and it is suggested to provide all individuals with emotional eating with an impulse control exercise complementary to their tailored exercises. Literature suggests that it is important to consider impulsivity-related personality traits for assessments and for treatment [78]. Paying attention to personality traits can improve clinical assessment, suggest points of intervention, and help tailor prevention and treatment approaches [79, 80]. As Gerlach et al. state “It is meaningful to identify subgroups of patients for whom specific treatment options need to be developed, such as measures for strengthening self-control skills” [81, p.33].

Quantitative results of the current study showed that the online body scan and positive reframing exercise may have contributed to an increase in positive affect and decrease in negative affect and overall emotion dysregulation within a sample of individuals with emotional eating. This study revealed small, but no significant changes in the expected directions. The changes did not reach statistical significance. Therefore, these small effects should be replicated in other samples, since we cannot rule out that the sample size and the large dropout in the current study has produced these results. While only minor changes in the expected directions were observed, participants clearly indicated which aspects contributed positively for them. It is important to explore these valuable starting points to further improve the tailoring of emotion regulation exercises for individuals with emotional eating, to meet specific emotion dysregulation.

Finally, it is noteworthy that 43% of participants have never consulted a dietitian. It is quite possible that they rely on their own knowledge and experience around dieting and are unaware of the potential added value of a dietitian, such as, for example, prescribing a diet so that they eat enough and whole foods throughout the day. In this way, a dietitian could be of added value for individuals with emotional eating, but because this group is ashamed of their eating behaviour, they are not likely to seek help. Providing online support could potentially remove obstacles in this regard.

### Strengths and limitations

A strength of the current study is the fact that it explored emotion regulation exercises in an online format. Participants reported positive experiences with it. This is threshold lowering because due to feelings of shame, individuals with emotional eating often are at distance from care (Evans) [81].

Another strength is the fact that participants scored high on emotional eating behaviour (M_*emo*_ = 3.48), corresponding to those of the emotional eater’s norm-group of obese women with ages between 21 and 40 years [59].

A limitation of the study is the fact that participants were asked their opinions about the online exercises at a late stage in the study. The open-ended questions were only asked "post-intervention" (T2), whereas more information could have been retrieved if the same questions had been asked at the start and during the study period. A large number of participants dropped out early and the reason for this is unknown.

It is particularly important to involve end users throughout the development process so that any problems in understanding or performing the exercises are recognized and resolved in a timely manner. Despite the fact that a pilot study was conducted, little feedback was received in regards to the problems experienced by the participants.

#### Allocation of participants

Another limitation is that, in contrast to what was expected, the allocation of participants based on specific emotion regulation difficulties resulted in two instead of three significantly different intervention groups. Because of this, group B was particularly small in sample size and the current study was unable to describe scientifically relevant effects of the opposite action exercise.

Due to its explorative nature, the current study did not include a control group without any online exercises. Therefore, the current study cannot assure that found effects would not also occur without the online tailored interventions.

#### Dropout

A remarkable phenomenon of the current study is the high dropout rate, from 80 participants at baseline (T0) to 15 participants at post-intervention (T2). On one hand, the online character of both the sampling strategies and the two-week training protocol can be seen as an important strength of the current study as it might have tackled one of the personal barriers for both seeking and completing face-to-face treatment in adults with emotional eating behaviour and overweight or obesity, namely: fear of stigma and shame [82]. This fear of stigma and shame could also explain why many participants in the current study (43.8%) have never had any contact with a dietitian before. However, the dropout in the current study was high and must be noted as an important limitation. At the end of the two-week training protocol dropout rates ranged from 60.0% for group B to 80.5% for group A, and 85.3% for group C. Overall, studies show a low adherence for internet-based interventions [83] and e-therapy [84] and the dropout rates in this study are similar to previous internet intervention dropout rates.

A wide array of factors might have influenced participants’ withdrawal. Since the departing participants were not interviewed about their leaving, it is not known why they stopped participating. Reasons could be the noticeable participant burden of practicing every other day for ± 15 min per session. The duration and schedule of the exercises was too extensive to adhere to in daily live. There has been an increase in boredom due to the lack of variation provided in exercises. Some participants did not (fully) understand the assignment. It might also have been the case that participants did not feel like being confronted with themselves repeatedly. Finally, participants may have picked up the desired knowledge and left. Annoyance arose about the amount of email reminders (eight in total) across the two-week training protocol. Participants suggested scheduling the exercise at varying time points in the day. They also indicated that the recurrence of practice moments was too close together. These aspects may also have played a role in the high dropout rates.

### Future research

Based on the study findings and literature of Murray and Vickers [77] and Tihanyi et al. [67] it is recommended for future research to study the supplementation of the body scan exercise. For example, the body scan exercise could be supplemented with an exercise component that prompts participants to pay attention to specific hunger sensations and help them distinguish these hunger sensations from emotionally evoked physical sensations as described above.

Another possibility would be to include a component in the exercise focused on making individuals with emotional eating aware of and helping them deal with automatic thoughts and judgements about their body or bodily sensations. The moment the user indicates negative associations with a bodily part, being in a vulnerable situation and susceptible to influences, a personalized virtual coach can make just-in-time matching suggestions to the situation with, for example, presenting a positive reframing exercise by applying JITAI’s.

Just-in-time adaptive interventions [85, 86] could, in conjunction with continuous monitoring and the deployment of Ecological Momentary Assessments (EMA’s) [87–89], provide the framework for real-time (or near-time) feedback that builds off prior knowledge of individuals in general and data collected previously on an individual. This compiled, dynamic knowledge can be used to provide information, nudges, and interventions when needed or appropriate, tailored to the individual’s needs and context, via mobile technologies [90]. This could help future users to decrease potential negative emotions, such as shame or fear, and uncomfortable attention-related body sensations such as muscle stiffness.

Factorial experimental design studies [91] can be performed to explore if the supplementation with these components increases the effectiveness of the body scan exercise within a sample of individuals with emotional eating. It is also quite possible that future factorial experimental design studies display limits to where individuals with emotional eating can effectively be supported by online personalized emotion regulation exercises and highlight where blended interventions with face-to-face support from health care professionals is needed [92].

#### The importance of context in tailoring

The current study aimed to spark advancements in future interventions for individuals with emotional eating by tailoring on individual emotion regulation difficulties. For two of the three groups (group A and C), this is promising, since it was recognized by participants as useful. However, to further improve the tailoring many more factors should be considered. For example, in the current emotion regulation literature, there is an increased focus on acknowledging the importance of the context when people select emotion regulation strategies [93]. In their study Tang and Huang showed that contextual factors, such as location (e.g., home, social or professional setting) and social context (e.g., alone or with others) could predict the selection of specific emotion regulation strategies. Accordingly, some participants in the current study, who practiced with the positive reframing exercise, did not consider the exercise for everyday use, and questioned its usefulness in varying circumstances because of its extensiveness. Subsequently, in accordance with the current emotion regulation literature, it is recommended for future research to use experience sampling methods [53] to identifying which contextual factors (e.g., location and/or social context) may predict the willingness of individuals with emotional eating to use emotion regulation exercises. The deployment of JITAI models could support this by tailoring specific emotion regulation strategies to the contextual factors at stake at that moment [86].

Deployment of EMA’s allow a personalized virtual coach to provide appropriate interventions at the right time. The decision rules to be applied can be based on dynamic computer models derived from EMA data according to the target behavioural variables, translating users’ input into tailored exercises.

#### Implications for future design

To decrease potential dropout, future work should focus on reasons for dropout, such as further reducing participant burden, avoiding boredom by providing variation in exercises, and optimizing the use of reminders. Users should be empowered by deployment of EMA’s to determine their own moments of practice and reflection. Applying JITAI’s can help identifying those moments.

Finally, it is important that when developing exercises, their presentation and the accompanying explanations better meet the needs of end users by involving them in the development process from start.

## Conclusions

In accordance with the discussion, we can conclude that the positive and negative experiences gained by users, and the resulting suggestions for improvement, can significantly improve the exercises and that modifications to be made, will have a positive impact on the effectiveness of the exercises.

Based on the current findings, we can cautiously suggest that further research among a larger population on delivering online exercises for this hard-to-reach group holds promise.

### Recommendations

Firstly, consideration should be given to the performance of the exercises, looking at frequencies and timing; based on events in their own context, users can better decide for themselves when to perform the exercises. Experience sampling methods could help to identify which contextual factors can play a role.

And lastly, based on the feedback of participants, it is recommended for future studies to employ additional factorial design studies to explore working components of online emotion regulation exercises further and experience sampling methods to explore the contexts in which these exercises could be most effective to assign to individuals with emotional eating.

#### Engagement

Dropout can be prevented by engaging and maintaining contact with participants during the development and use of exercises by providing feedback and explanations of the exercises where needed and desired. It is important that instructions to participants are engaging so that participants engage in the exercises. It is important that participants are enabled to share their user experiences, so that valuable information will not get lost because participants have dropped out early.

Finally, it is important that when developing the exercises, their presentation and the explanations required, better reflect the needs of the end users. They should be involved in the design process from the first steps of intervention development.

#### Tailoring

The current study encourages explorative research on the development of tailored interventions for individuals with emotional eating and contributes to the previous study of Dol et al. [36] with the recommendation to include tailoring for difficulties in emotion regulation in the development of a personalized online coach.

Overall, the current study was able to provide valuable starting points to further improve the tailoring of emotion regulation exercises for individuals with emotional eating and to subsequently spark advancements in the online just-in-time treatment of emotional eating behaviour.

## Figures and Tables

**Fig. 1:**
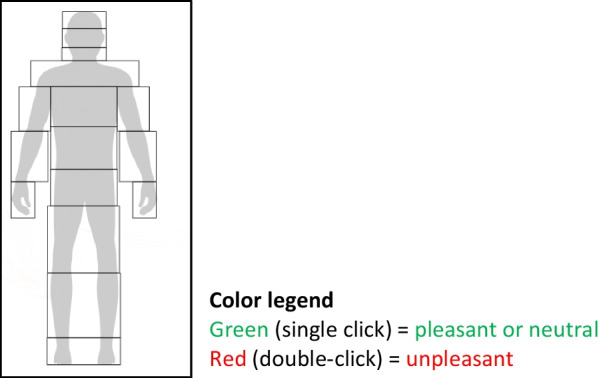
Schematic overview body

**Fig. 2:**
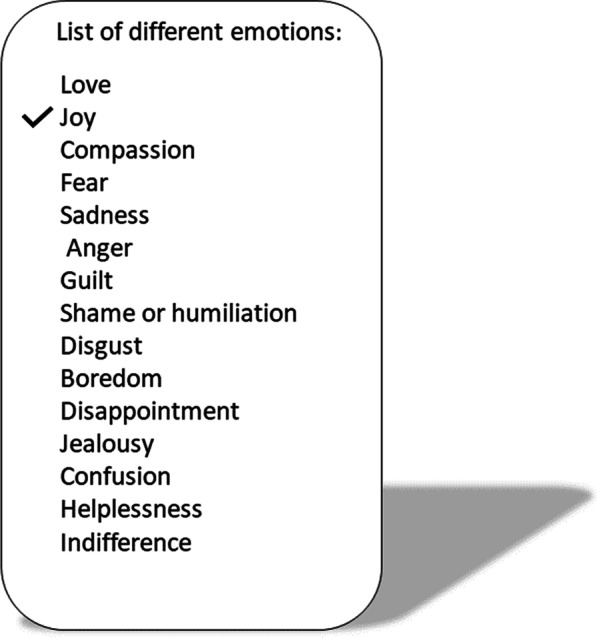
List of emotions

**Fig. 3:**
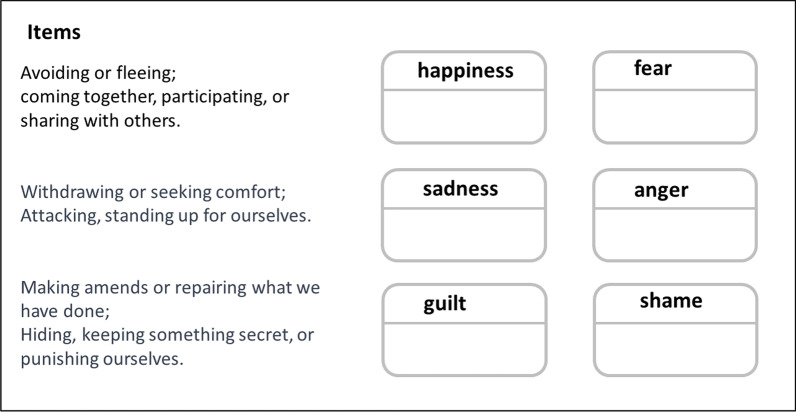
Input fields

**Fig. 4:**
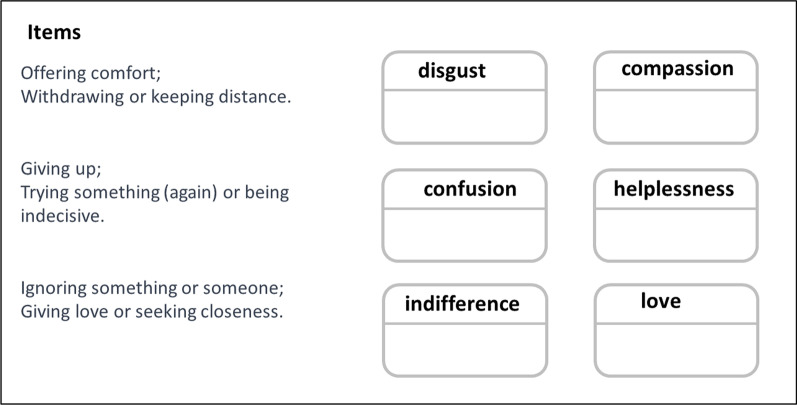
Input fields

**Fig. 5:**
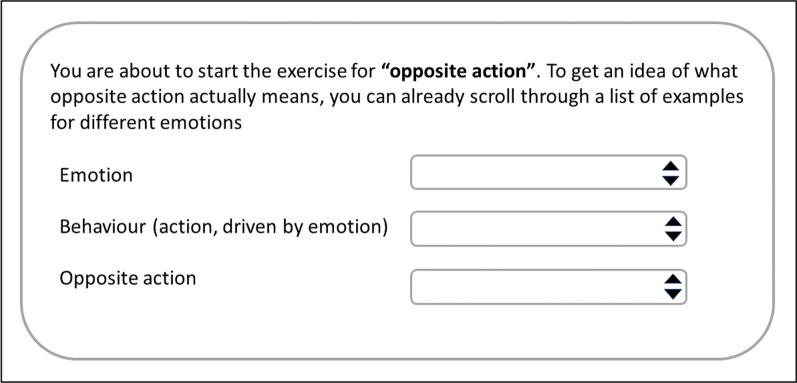
Input fields

**Fig. 6:**
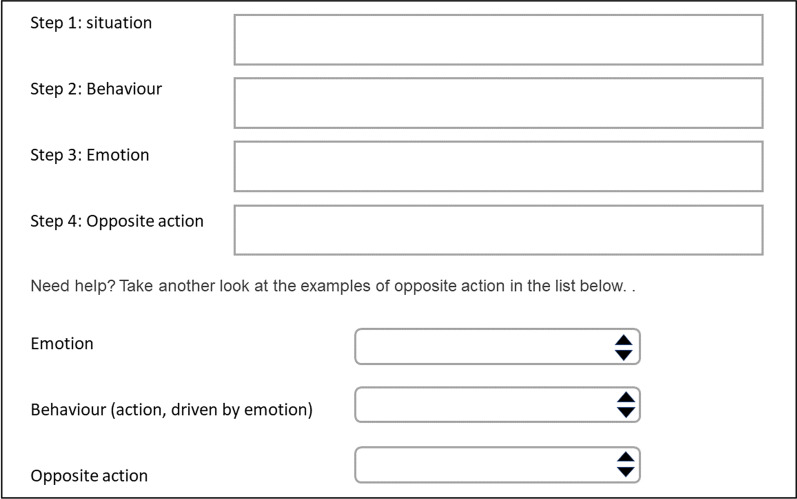
Input fields

**Fig. 7:**
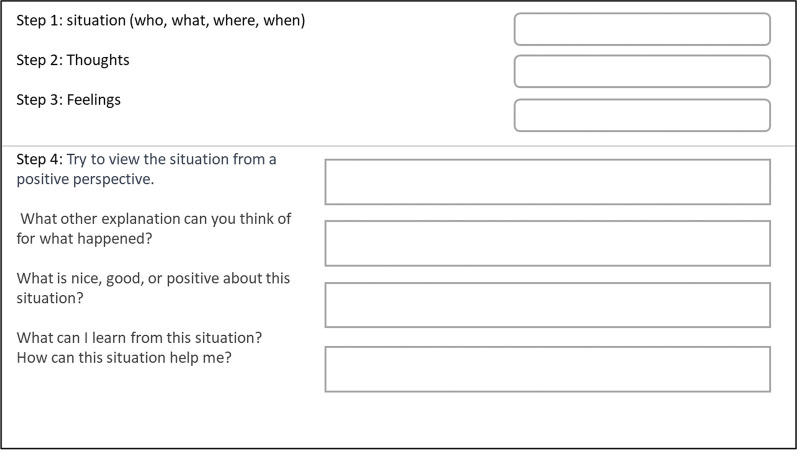
Input fields

## Data Availability

The three exercises and qualitative datasets (i.e. transcripts, codes, and themes) used and/or analyzed during the current study are available from the corresponding author upon reasonable request.

## Appendix 1: Body scan exercise

Dear participant,

Welcome to the **"body scan exercise".**

The purpose of this exercise is to make you more aware of what you feel in your body. It also encourages you to reflect on the role that emotions can play in these sensations. The interesting thing is that our body can tell us a lot about the emotions we experience. For example, think about your heart pounding in your chest when you’re in love, or the sweat on your hands when you’re nervous for a job interview.

Often, we find it easier to notice sensations in our body than to recognize our emotions. Therefore, the "body scan exercise" first invites you to pay attention to what you feel in your body and then to consider what may have caused these sensations (e.g., a certain emotion).

The exercise consists of two challenges and lasts a maximum of 15 min.

Good luck and enjoy the practice!

**Challenge 1: Scan your body**

Step 1: Examine the different areas of your body in the image below.

Step 2: Take a moment to focus on each body area for approximately 30 s.

Step 3: Indicate for each body area whether it feels pleasant/neutral (by clicking once on the body area) or unpleasant (by double-clicking on the area) (Fig. 1).

**Challenge 2: Find an explanation**

Step 1: Reflect on the "body scan exercise".

Step 2: Consider a possible explanation for what you felt in your body.

Step 3: Respond to the statement below.

What I felt in my body can be explained by:

(note: Your own experience is central. Every answer is correct and multiple answers are possible).

- Physical processes, namely __________________________.
  (example: hunger or thirst, muscle pain, symptoms of illness).
- Emotion, namely __________________________________.
  (example: see list of different emotions below).
- Other, namely ____________________________________.

See Fig. 2

## Appendix 2: Opposite action exercise

Dear participant,

Welcome to the **"opposite action exercise".**

You may have noticed that your emotions influence your behaviour. Sometimes in a positive way, for example: you see spoiled food→you feel disgust→you keep your distance→you don’t eat it→you don’t get sick.

However, emotions are not always the perfect guide. It may happen that you have been overwhelmed by a certain emotion and have done something you may not have wanted to do. One strategy to prevent this and reduce a specific emotion is to change the behaviour that triggers the emotion. Acting in a way that is opposite to the emotion can be helpful.

The opposite behaviour exercise aims to help you:

1. become aware of your own behaviour,
2. reflect on which emotion has influenced your behaviour, and.
3. regain control over your behaviour by practicing opposite behaviour.

The exercise consists of two challenges and will take up to 15 min. If you would like to practice recognizing behaviour that can stem from different emotions, start with a brief introduction exercise.

Good luck!

- Start with the introduction exercise!
- Begin directly with the opposite action exercise!

**Do the introduction exercise and discover how emotions influence our behaviour!**

Drag the ‘behaviour’ to the appropriate emotion (Note: each behaviour fits only one of the two emotions).

See Figs. 3, 4 and 5.

**Opposite Action Exercise**

**Challenge 1**

- Step 1: Think about a **situation** you experienced today and describe it.
- Step 2: Describe the **behaviour** you exhibited in this situation.
- Step 3: Reflect on which **emotion** could have influenced your behaviour and describe this emotion.
- Step 4: Think of and describe behaviour that is the **opposite** of your behaviour in step 2.

Fill in your answers here:

See Fig. 6

**Challenge 2**

Now that you know which behaviour can help reduce the emotion (see step 4), try it out in a similar situation (or with a similar emotion) by consciously practicing the opposite behaviour and observe how it affects your emotion.

Tip: Don’t be too hard on yourself if it doesn’t work immediately. Be proud of the fact that you tried and give it another try another time.

Best of luck!

## Appendix 3: Positive reframing exercise

Dear participant,

Welcome to the **"reframing exercise".**

This exercise will challenge you to look at a difficult situation from a positive perspective. How we feel is strongly influenced by how we think about a particular situation. Consider these two examples:

- Example 1: You have a conflict with your partner→You think: "Ugh, I hate him! I wish he would leave."
- Example 2: You have a conflict with your partner→You think: "Well, maybe he had a rough day at work."

Did you notice the difference? It’s likely that you feel more positive after reading example 2.

With the positive reframing exercise, you can now practice daily reinterpreting challenging situations. The exercise will take a maximum of 15 min.

Good luck and enjoy practicing!

**Positive Reframing Exercise**

Step 1: Describe the challenging **situation** (Who was involved? What happened? Where and when did it occur?).

Step 2: Describe your **thoughts** (What thoughts were going through your mind?).

Step 3: Describe how you **felt** (What emotion(s) did the situation evoke in you?).

Step 4: Try to look at the situation from a **positive perspective**.

Fill in your answers here:

See Fig. 7

**Questions & Answers**

1. **I haven’t experienced any challenging situations today, what now?**
  If you still want to practice, you can also use a situation that occurred a while ago. The more you practice, the easier it becomes to break old thinking patterns.
2. **I’m having trouble answering all the questions in step 4, what now?**
  That’s not a problem! The questions are there to challenge you to look at a situation differently. It’s possible that it doesn’t work as well for every situation. Even if you can only answer one out of the four questions, it means you have already taken the first step towards breaking your old thinking patterns. This is not easy. You can be proud of yourself!
3. **The situation I experienced is too personal. I prefer not to answer the questions in the OMdenk exercise via the survey, what now?**
  It’s good to know that your answers in the OMdenk exercise will not be used for research purposes. However, you can always choose not to answer the questions in the survey. Perhaps it feels more comfortable for you to do the exercise with pen and paper? The most important thing is that you do the exercise in a way that feels right for you.

## Footnotes

1 All example sentences have a code referring to the corresponding exercise and a sequence number.
